# An in-silico layer-by-layer adsorption study of the interaction between Rebaudioside A and the T1R2 human sweet taste receptor: modelling and biosensing perspectives

**DOI:** 10.1038/s41598-020-75123-4

**Published:** 2020-10-27

**Authors:** Olayide A. Arodola, Suvardhan Kanchi, Phathisanani Hloma, Krishna Bisetty, Abdullah M. Asiri, Inamuddin

**Affiliations:** 1 Department of Chemistry, Durban University of Technology, P.O Box 1334, Durban, 4000 South Africa; 2 Chemistry Department, Faculty of Science, King Abdulaziz University, Jeddah, 21589 Saudi Arabia; 3 Advanced Functional Materials Laboratory, Department of Applied Chemistry, Faculty of Engineering and Technology, Aligarh Muslim University, Aligarh, 202 002 India

**Keywords:** Carbon nanotubes and fullerenes, Electrochemistry

## Abstract

The human sweet taste receptor (T1R2) monomer—a member of the G-protein coupled receptor family that detects a wide variety of chemically and structurally diverse sweet tasting molecules, is known to pose a significant threat to human health. Protein that lack crystal structure is a challenge in structure-based protein design. This study focused on the interaction of the T1R2 monomer with rebaudioside A (Reb-A), a steviol glycoside with potential use as a natural sweetener using in-silico and biosensing methods. Herein, homology modelling, docking studies, and molecular dynamics simulations were applied to elucidate the interaction between Reb-A and the T1R2 monomer. In addition, the electrochemical sensing of the immobilised T1R2-Reb-A complex with zinc oxide nanoparticles (ZnONPs) and graphene oxide (GO) were assessed by testing the performance of multiwalled carbon nanotube (MWCNT) as an adsorbent experimentally. Results indicate a strong interaction between Reb-A and the T1R2 receptor, revealing the stabilizing interaction of the amino acids with the Reb-A by hydrogen bonds with the hydroxyl groups of the glucose moieties, along with a significant amount of hydrophobic interactions. Moreover, the presence of the MWCNT as an anchor confirms the adsorption strength of the T1R2-Reb-A complex onto the GO nanocomposite and supported with electrochemical measurements. Overall, this study could serve as a cornerstone in the development of electrochemical immunosensor for the detection of Reb-A, with applications in the food industry.

## Introduction

The two steviol glycosides, rebaudioside A and stevioside, are natural sweeteners extracted from Stevia *rebaudiana* which belongs to the Asteraceae family^1^. Stevia sweeteners are obtained from the dried stevia leaves and the extracts are popularly used as a non-caloric substitute for sugar. Stevia contain steviol glycosides that are approximately 150–300 times sweeter than sugar. This plant-based sweetener is also believed to have a positive effect on blood sugar levels, with steviol glycoside as one of the natural sweeteners responsible for the increased blood sugar level in most diabetic patients^2^. Furthermore, the potential of steviol glycosides to induce sweet and bitter taste sensations have been reported^3^, however, its bitter aftertaste has long prevented it from becoming widely marketed as food and beverage sweeteners^4^.

Stevia extracts, besides having therapeutic properties, contain a high level of sweetening compounds known as steviol glycosides, which possess antioxidant, antimicrobial and antifungal activities^5^. It has also been reported that rebaudioside A (Reb-A) are mostly used as food additives for dairy products, confectionaries and beverages^6,7^. Most of the artificial sweeteners are low-calorie sweeteners responsible for some of the health-related threats posed by sucrose. The stability of these sweeteners at even higher temperatures have increased their presence in food application. Although there are no reports of stevia glycosides on the health risks of steviol glycosides, its widespread applications in the food industry needs to be regulated.

**Figure 1:**
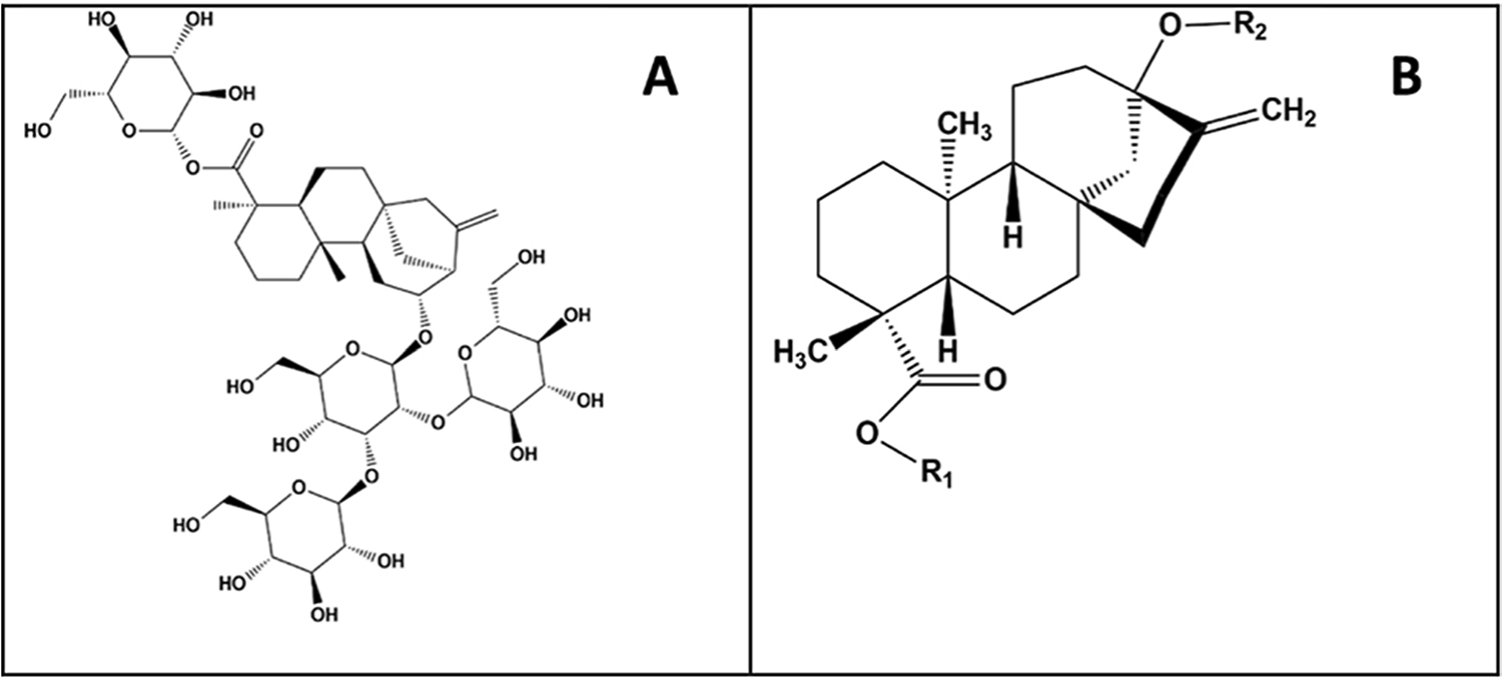
Chemical structures of (**A**) Rebaudioside A (Reb A) and (**B**) Stevioside (Stv).

Reb-A is a diterpene glycoside, which contains a total of four glucose molecules wherein the central glucose is connected to the main steviol backbone, and the remaining glucose at its carboxyl group end, forming an ester bond (Fig. 1A), with 13 rotatable bonds, 14 hydrogen bond donors, and 23 hydrogen bond acceptors. On the other hand, Stevioside (Stv) is a glycoside consisting of the aglycone steviol and three glucose molecules (Fig. 1B)^7^.

The human sweet taste receptor (STR) is a heterodimer consisting of the T1R2 and TIR3 subunits which belongs to the class C G-protein coupled receptor (C-GPCR) family. Sucrose, glucose, sucralose and related sugars were reported to interact with the active sites of the amino terminal domain (ATD) of both T1R2 and T1R3 subunits^8,9^. For example, the dipeptidyl sweeteners such as aspartame and neotame appear to find a binding site in the T1R2 N-terminal domain, which can be considered as orthosteric agonists^10,11^. Similarly, Neoculin and brazzein are thought to interact with the N-terminal domain^12^ and the cysteine-rich area^13^ of human T1R3, respectively.

To date, few studies have reported the modelled structure of the T1R2 and T1R3 subunits of the STR family for comparative activities of the sweeteners^10,14^. However, the interaction between Reb-A and proteins such as bovine serum albumin, hT2R4 and hT2R14 bitter taste receptors have also been reported^15,16^.

Although the development of a cytochrome C biosensor for the detection of Reb-A has been previously reported in our laboratory using electrochemical and computational methods^17^, to the best of our knowledge, the development of a human T1R2 biosensor for the detection of Reb-A is yet to be reported in literature. In this study, the selection of a member of the T1R family as a candidate taste receptor was based on their physiology of taste perception embodied in the characteristics of T1R function. The T1R2/T1R3 receptor has a broad ligand specificity, recognizing monosaccharides to oligosaccharides, artificial sweeteners without saccharide groups, some D-amino acids and even proteins. However, T1R3 undergo mutagenesis and resulting in the lowering of its activity due to cysteine-rich domain of T1R3, but not in the case of T1R2^18^. Therefore, T1R2 receptor has been chosen as a selective bio-recognition element for effective sensing platform. Accordingly, computational tools were employed to assess the feasibility of using T1R2 receptor in the development of an electrochemical immunosensor with unique features including higher sensitivity and selectivity.

Literature studies revealed that diverse analytical methods have been employed in the food industry to quantify steviol glycosides, such as Reb-A. Some of these instruments include liquid chromatography-mass spectrometry^19,20^, high performance liquid chromatography (HPLC)^21,22^, high performance thin layer chromatography^23^, and capillary electrophoretic methods^24,25^, among others^26^. In contrast, noteworthy advantages such as low cost, higher sensitivity and selectivity with quick response times, are attributed to electrochemical methods. One such method was reported by Komorsky-Lovrić et al.^27^. However, the non-availability of the human T1R2 crystal structure or the lack of information about its mechanism at an atomistic level may have influenced the lack of a human T1R2 immunosensor for the detection of Reb-A in literature. Therefore, this is a fertile ground for research and it is deemed timely to develop an immunosensor for the detection of Reb-A that have improved attributes such as better selectivity, sensitivity, robustness and first-rate catalytic activity.

Recently, nanomaterials have been employed in the modification of electrodes due to their recognized sensitive, reliable and selective determination of organic compounds. Accordingly, the incorporation of multiwalled carbon nanotubes (MWCNTs) could prove useful as a result of their widespread capabilities in sensing applications^28^ when used for the analysis of a wide range of analytes^29–32^. Generally, the surface sensitivity of MWCNTs further confirms their choice as an ideal material for nanoscale biosensor devices that require high sensitivity^33,34^. Similarly, graphene oxide (GO) is commonly used due to the attachment of the –OH, –COOH and –C–O–C– functional groups to its plane^35^. More recently, GO was used for the fabrication of biosensors such as the detection of organic molecules with pharmaceutical, food and environmental importance^35–37^.

Likewise, ZnONPs, being one of the most important metal oxide nanoparticles, are popularly employed in diverse fields due to their unique physical and chemical properties^38,39^. ZnONPs have attracted significant attention in the development of electrochemical biosensors^39–41^. This is due to their unique properties such as a suitable micro-environment for the immobilization/loading of enzymes and proteins while retaining their biological activity, electronic properties supporting different device categories, low cost, ease of preparation, biocompatibility, and catalytic surface activity. Thus, this has prompted their expanded use in the construction of electrochemical biosensors with enhanced analytical performance^41–44^. Thus far, the immobilization of two types of nanovesicles, which have human T1R2/T1R3 for the umami taste and sweet taste on their membranes, onto graphene surfaces for the simultaneous detection of umami and sweet tastants have been reported^45^. However, the immobilization of human T1R2 onto GO in the presence of ZnONPs and MWCNT for the detection of Reb-A is still unreported in literature.

Hence, the motivation and the need to use in-silico approaches, which have been shown to rapidly and accurately estimate the activities and properties of nanoparticles^46,47^, reduce costs and to determine predictive variables that can be used in guiding wet lab experiments^48^. In-silico methods accurately describe the molecular surface interactions for a better understanding of the reactions on surfaces. Surface studies are essential because they offer useful insights on the surface reactivity with likely changes of the nanoparticles, due to interactions with different elements^49^.

Herein, we provide information on the interaction between Reb-A and human T1R2 monomer as well as their physical interactions with MWCNT, ZnONPs and GO through the application of an array of methodologies such as homology modelling, molecular docking, molecular dynamics simulations, adsorption studies, and electrochemical biosensing. This study could serve as a cornerstone in the development of electrochemical immunosensor with enhanced selectivity and better performance.

## Computational models and methods

### Template selection

Due to the absence of a crystal structure of the human taste receptor 1 (T1R2), the 3D structure was modelled using the human taste receptor type 1 member 2 sequence obtained from Uniprot (Uniprot ID: Q8TE23) as template. Homology modelling was performed using the Chimera software^50^ as an interface to the Modeller Software version 9.1^51^. The model was validated using the Ramachandran plot of the Schrodinger Maestro Suite^52^ and the active site residues were determined using the Site-Hound web program^53^.

The sequence of the target protein was uploaded unto PSIPRED V3.3^54,55^ in order to obtain a predicted 3D secondary structure of the human T1R2. The assessment of the bond angles and torsional strain shows the evaluation of the predicted 3D structure. A Ramachandran plot for the analyses of bond angles and torsional strain was generated using Maestro^52^. MolProbity^56^ result shows 86.7% (726/837) of all residues were present in favoured (98%) regions, while 94.9% (794/837) of all residues were in the allowed (> 99.8%) regions, with 5% (43/837) as outliers. The list also confirms that none of the active site residues were part of these outliers.

### Molecular docking of the human T1R2-Reb-A complex

The 3D structure of Reb-A was retrieved from the PubChem database^57^ and docked onto the human T1R2 monomer homolog using Autodock Vina^58–61^. This software performs the prediction of the bound conformation based on free energy, calculated on the basis of the empirical force field and the Lamarckian Genetic Algorithm^62^. The Grid module was used to create a grid box of dimensions X = 56, Y = 70 and Z = 56 along the center X = 14.542, Y =  − 16.714 and Z = 63.662 with a spacing of 0.375 Å. The efficiency of the predictions was amplified by setting the parameters associated with the Lamarckian genetic algorithm to the maximum efficiency values. The docked T1R2-Reb-A conformation with the highest docking score was selected for the molecular dynamics (MD) simulations. The resulting human T1R2-Reb-A complex was used for all subsequent methods performed in this study.

### Molecular dynamics (MD) simulations

All atom molecular dynamics simulations have been performed on the docked complex using the AMBER 18 molecular dynamics package^63,64^. The bonded and non-bonded description of the interactions between the various atoms have been described using the AMBER18 force fields which include the GAFF^65^ force field parameters. Due to the lack of parameters needed for Reb-A in the Cornell et al. force field^66^, the missing parameters were generated. Optimization of the Reb-A was performed at the HF/6-31G* level with the Gaussian 09 package^67^. The human T1R2 homolog was energy minimized and equilibrated via molecular dynamics simulations and discussed as follows. The standard AMBER force field for bioorganic systems (ff03)^68^ was used to define the topology and parameter files for the T1R2 homolog and Reb-A using “GAFF”^65^ force field parameters based on the atom types of the force field model developed by Cornell et al.^63^. The topology and coordinate files were generated using the antechamber module of AMBER 18, and the missing hydrogen atoms were added and saved for the T1R2 homolog using the LEaP module in AMBER 18. Thereafter, the docked T1R2-Reb-A complex was solvated in the octahedron box of TIP3P water model^64^ with buffering distance of 10 Å between the protein surface and the box boundary as well as the neutralization was performed by adding 9 Na^+^ counterions to mimic the biological pH. Cubic periodic boundary conditions were imposed, and the long-range electrostatic interactions were treated with the particle-mesh Ewald method implemented in AMBER 18 with a non-bonding cut-off distance of 10 Å.

Initially, a series of energy minimization steps were performed to eliminate any bad contacts in the initially built structures while restraining the solute with 500 kcal/mol/Å^2^ harmonic force constant. The above system was minimized for 1000 cycles of steepest descent followed by 2000 cycles of conjugate gradient. The entire system was freely minimized for 1000 iterations. Heating was performed for 50 ps from 0 to 300 K with harmonic restraints of 5 kcal/mol Å^2^ using a Langevin thermostat with a coupling coefficient of 1/ps. The entire system was then equilibrated at 300 K with a 2 fs time step in the NPT ensemble for 500 ps and Berendsen temperature coupling^69^ was used to maintain a constant pressure at 1 bar. The SHAKE algorithm^70^ was employed on all atoms so as to constrain the bonds of all hydrogen atoms.

With no restraints imposed, a production run was performed for 100 ns in an isothermal isobaric (NPT) ensemble using a Berendsen barostat with a target pressure of 1 bar and a pressure-coupling constant of 2 ps for analysis of the energy stabilization and RMSD values of the complex. The coordinate file was saved every 1 ps and the trajectory was analyzed every 1 ps using the Ptraj module implemented in AMBER 18.

The thermodynamic property of the system was calculated. The free binding energy of Reb-A to the human T1R2 homolog active site was analysed by the Molecular Mechanics/Generalized Born Surface Area (MM/GBSA) method^71–73^. A single trajectory approach was used with 100,000 snapshots at 100 ps interval of each simulation. From each snapshot, free binding energy (ΔG_bind_) was computed from the following equation:

1 $$ \Delta {\text{G}}_{bind} = {\text{ G}}_{{hT1R2{ - }Reb{ - }A}} {-}{\text{ G}}_{hT1R2} {-}{\text{ G}}_{{Reb{ - }A}}$$

where ΔG_*bind*_ is the free binding energy; G_*hT*1*R2-Reb-A*_ is the energy of the human T1R2-Reb-A complex; G_*hT*1*R2*_ is the energy of the human T1R2; and G_*Reb-A*_ is the energy of the Reb-A. Due to the high computational cost in the entropy calculation, 100 snapshots were extracted from the last equilibrated 100 ns trajectory of the simulation with 100 ps time intervals.

To theoretically evaluate the reliability of the calculated ΔG values, the standard error (SE) of the calculated free binding energy was estimated by using Eq. (2), which is related to the number (N) of snapshots chosen for the calculations^60^.

2 $$ {\text{SE }} = {\text{ RMSF}}/{\text{N}} $$

### Nanomaterials modelling

All studied nanoclusters were built using Materials Studio (MS) BIOVIA^74^. Geometry optimization of the nanomaterials were performed with the Forcite module^75^ as implemented in the MS software. Forcite in the MS software is a classical molecular mechanics tool, designed to perform a range of tasks including fast energy calculations and geometry optimizations for single molecules as well as periodic systems. A detailed knowledge of surface interactions play a key role in the design of many materials and processes. An important first step in the preparation of a model of molecules adsorbed onto the surface, is to ensure that the geometries are fully optimized. Among the different steps involved in the modelling approach are the construction of the surface from the pure crystal, the addition of the molecules near the surface, the selection of an appropriate forcefield to study the nanomaterial interaction, followed by initial calculations of the energy and geometry optimization. Modelling and visualization were carried out with VMD^76^, GaussView^77^, and Materials studio (MS)^74^.

Herein, a 3-D model of the GO surface comprising of a pristine graphite structure, conforming to the standard structural database in the MS software was built with oxygen atoms manually added. Likewise, a 3-D model of ZnONPs was built to conform to the standard structural database in MS. A supercell structure (6 × 6 × 1) of ZnO was built and the symmetry was set to have a non-periodic boundary. The unit cell of the bulk ZnO (110) was geometry optimized using the Forcite module, with a 8 × 8 × 8 Monkhorst-Pack^78^ mesh k-points and a kinetic energy cut-off of 4.5 Å. The unit cell was then relaxed using the conjugate gradient method until the total forces acting on each atom were 8.401e−04 kcal/mol/A. The ZnO (110) surface was geometry optimized by solving the Kohn–Sham equation self-consistently under spin-unrestricted conditions^79^. The double-layered MWCNT (n = 10, m = 10) was built using the Build option in the MS software. To observe the feasibility of all structures, their primitive structures were optimized by Forcite module with fine-COMPASS force field^75^. The convergence criteria for the maximum values of energy alteration, force, stress, and displacement were set at 2 × 10^−5^ kcal/mol, 0.001 kcal/mol/Å, 0.001 GPa, and 10^−5^ Å respectively. Frequency calculations were carried out to determine if the compounds are at the lowest surface energy and to deduce physical interaction energies i.e. adsorption.

Next, the Adsorption Locator (AL) module as implemented in the MS software was used as a preparatory and screening tool with the forcefield method to obtain a ranking of the energies for each generated configuration, thereby indicating the preferred adsorption sites. Since the adsorbate can be adsorbed at different locations on the GO surface, the AL module was used to deduce the best adsorption site with the lowest energy on the surface. The AL applies Monte Carlo simulations within a lattice dynamics approach to obtain the best configuration. The generated adsorbate–substrate conformations for GO/ZnONPs/T1R2-RebA and GO/MWCNT/ZnONPs/T1R2-RebA were ranked and the lowest adsorption energy conformers were each optimized using the Forcite-Geometry optimization to reach a stable conformation as shown in Table 1.

**Table 1:** Optimised geometries of nanomaterials used along with the minimized energies.

Nanomaterial	Optimized 3-D structures	Total optimized energy kcal/mol
GO		491.239
MWCNT		221,045.658
ZnONPs		288.891

The adsorption energies were calculated using the following equation:

3 $$ {\text{E}}_{{{\text{ads}} }} = {\text{ E}}_{{{\text{adsorbate}}}} + {\text{ E}}_{{{\text{surface}}}} {-}{\text{ E}}_{{{\text{adsorbate}}/{\text{surface}}}} $$

where E_adsorbate_ is the energy of the adsorbate energy without the surface, E_surface_ is the surface energy of the ZnO nanoparticles and E _adsorbate/surface_ is the total energy of the surface and the adsorbate. In this case, if E_ads_ < 0; the adsorption energy implies stability and the chemical process is exothermic, with a more negative value implying a stronger adsorption energy.

### Experimental method

#### Procedure for the fabrication of Pt-E with nanocomposite *GO/ZnONPs*

In this study, ZnONPs were synthesised from zinc gluconate using hydrothermal method. Then the GO solution was prepared according to Bathinapatla et al*.*^17^, by quantitatively measuring 1 ml of GO into 10 ml of deionized distilled water. The ZnONPs/GO nanocomposite was achieved by mixing ZnONPs dispersion and GO dispersion in a ratio of 1:1 and ultrasonicated for 1 h to obtain a homogenized mixture. T1R2 which serves as a biological recognition element for Reb A was drop-casted on the surface of GO modified Pt-E and left to stand at room temperature for 1 h before analysis. The finally prepared electrode was utilized to detect Reb A at lower concentration.

#### GO/MWCNTs/ZnONPs

To evaluate the significance of MWCNTs as an effective adsorbent, DMF was used as a dispersion medium. 0.5 g of MWCNTs was dispersed in a 2.5 ml of DMF and ultrasonicated for 1 h to make a thick solution. Then ZnONPs nanoparticles were added to the MWCNTs dispersion and sonicated at room temperature for 1 h to complete physio-adsorption of ZnONPs onto the surface of MWCNTs. This nanocomposite was coated on the Pt-E modified GO. Finally, T1R2 subunits were immobilized on the Pt-E/GO/MWCNTs to evaluate the efficiency of adsorbent in accommodating the Reb A.

#### Electrochemical evaluation of interaction of Reb-A with T1R2/ZnONPs/MWCNTs/GO/Pt-E

Electrochemical measurements to predict the interaction of Reb A with T1R2/ZnONPs/MWCNTs/GO/Pt-E was performed with differential pulse voltammetry studies at room temperature using three-electrode system electrochemical cell (VA797 Computrace, Metrohm, South Africa). The three-electrode system consist of platinum as the working electrode, graphite electrode as the counter electrode, and silver/silver chloride as the reference electrode. For measurements, the 20 ml electrochemical cell was filled with 10 ml of 0.1 M pH 11.0 borate buffer, 20 µ/of 100 mg/l of Reb A working standard.

## Results and discussion

### Modelling of the human sweet T1R2 taste receptor

**Figure 2:**
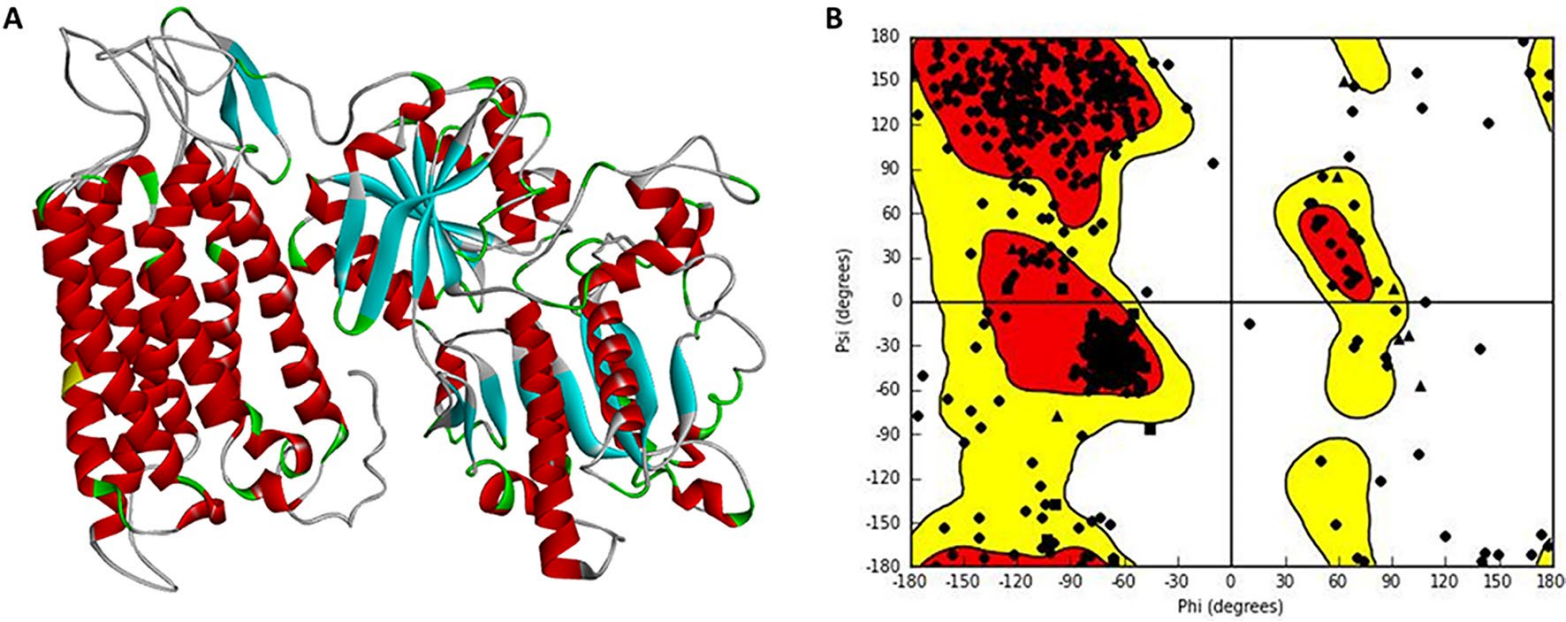
(**A**) Homology model of the Human Taste Receptor (T1R2) containing 839 amino acid residues (Image generated on Discovery studio v2016^80^ license accessed through CHPC, Cape Town) (**B**) The Ramachandran Plot showing the validation of the T1R2 monomer (Image generated using the Schrodinger Maestro Suite v2018^52^) .

The generated 3-D structure of T1R2 monomer (Fig. 2A) was further subjected to optimization tools for energy minimization and the active site residues were determined and validated using the Site-Hound web program^53^. Thereafter, the stereo-chemical qualities of the predicted structures were assessed using the Ramachandran plot (Fig. 2B) in Maestro Schrodinger suite^52^, affirming the validation of the homolog.

Closest active site residues were selected and used for further modelling studies. The obtained active site residues for the human T1R2 homolog are Thr183, Pro185, Ile327, Gln328, Ser329, Val330, Arg383, Val384, Tyr386, Ser387, His434, Gln435, Ile436, Phe437, Val444, Ala445, His447, and Arg837. Gln328, Asp433, His434, His447, Tyr469, Gln472, Leu575, Arg837, Arg838, Asp839. The MolProbity^56^ model validation show 86.7% (726/837) of the residues are in the favoured regions, while 94.9% (794/837 of the residues are in the allowed regions, with 5% (43/837) of the residues as outliers. This list confirms that none of the active site residues reported in this study are part of the outliers (see Supplementary Material). Thus, the above analysis serves as a basis that the predicted 3D structure of the human T1R2 monomer is of a good quality and can be used in this study.

### Molecular docking

**Figure 3:**
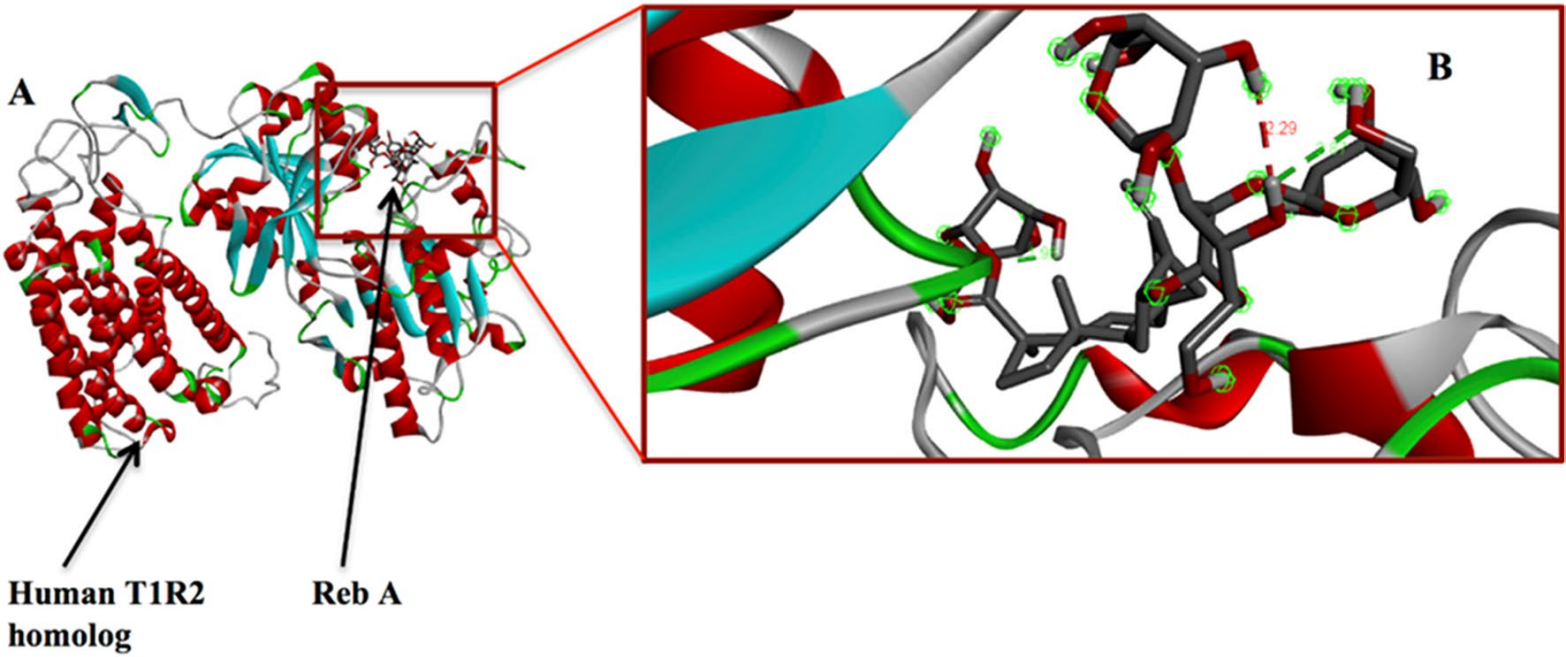
(**A**) The docked structure of the T1R2-Reb-A complex. (**B**) The Reb-A interaction with the human T1R2 monomer showing the distances between the active site residues and Reb-A in green and red colours, corresponding to a binding affinity of − 8.7 kcal/mol. (Images generated on Discovery studio v2016^80^ license accessed through CHPC, Cape Town).

As docking is the most appropriate tool to provide a better description of the binding theme of inhibitors^81,82^, molecular docking was performed to elucidate the binding modes of the human T1R2 and Reb-A complex. To this end, the binding affinity energy with 0 Å distance of deviation from the active site was − 8.7 kcal/mol (Fig. 3A,B).

**Figure 4:**
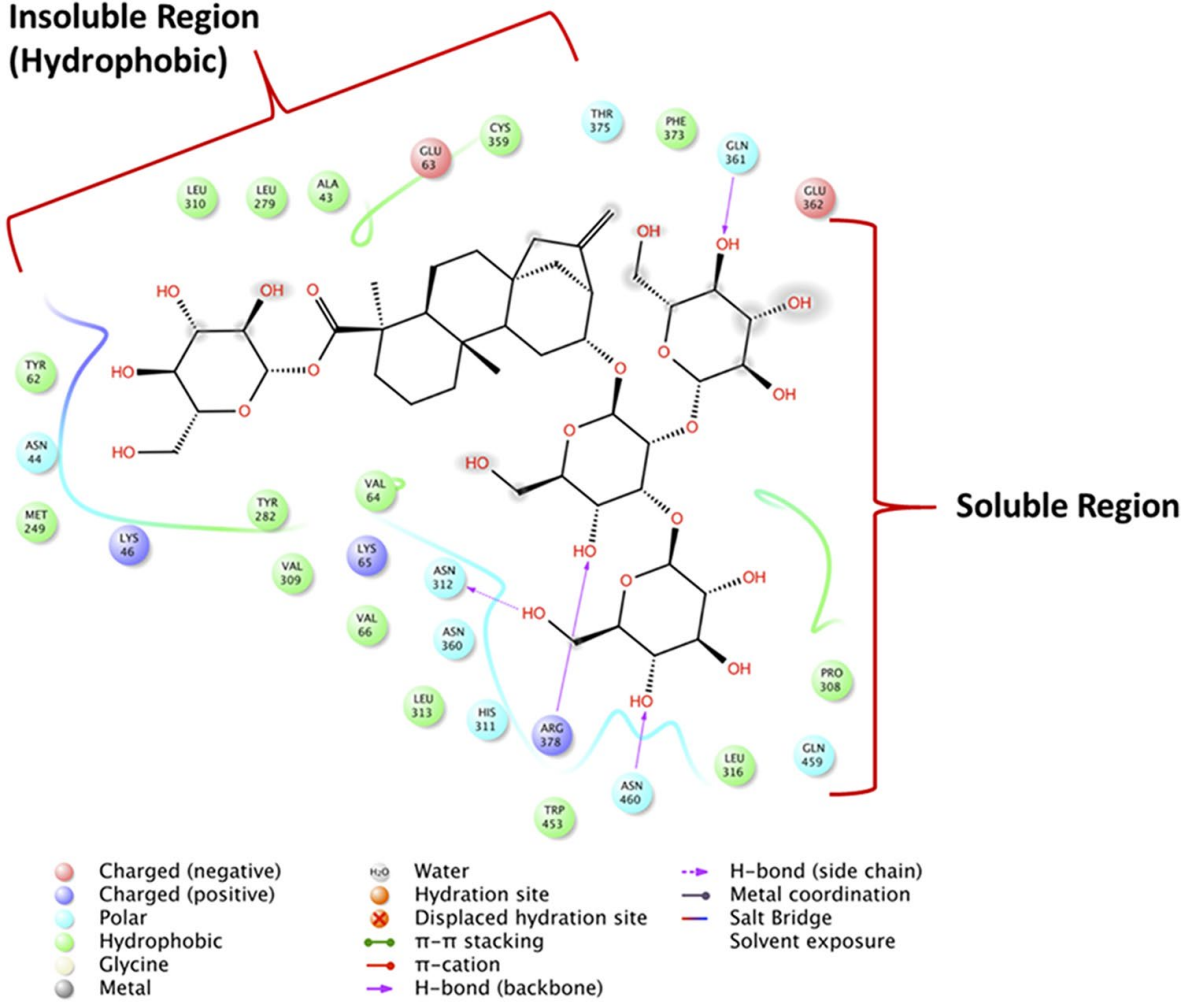
Reb A-T1R2 complex interaction at the predicted binding sites. Major contributions are from the residues that exhibit hydrophobic interactions (in green bubbles), corresponding to a binding affinity of − 8.7 kcal/mol (Image generated using the Schrodinger Maestro Suite v2018^52^).

Figure 4 shows the binding modes of Reb-A with the active site residues of the T1R2 receptor depicting major hydrophobic interactions from the active site residues such as Ala43, Tyr62, Val64, Val66, and Met249; hydrogen bond interactions from Asn312, Asn361, Arg378, and Asn460.

**Figure 5:**
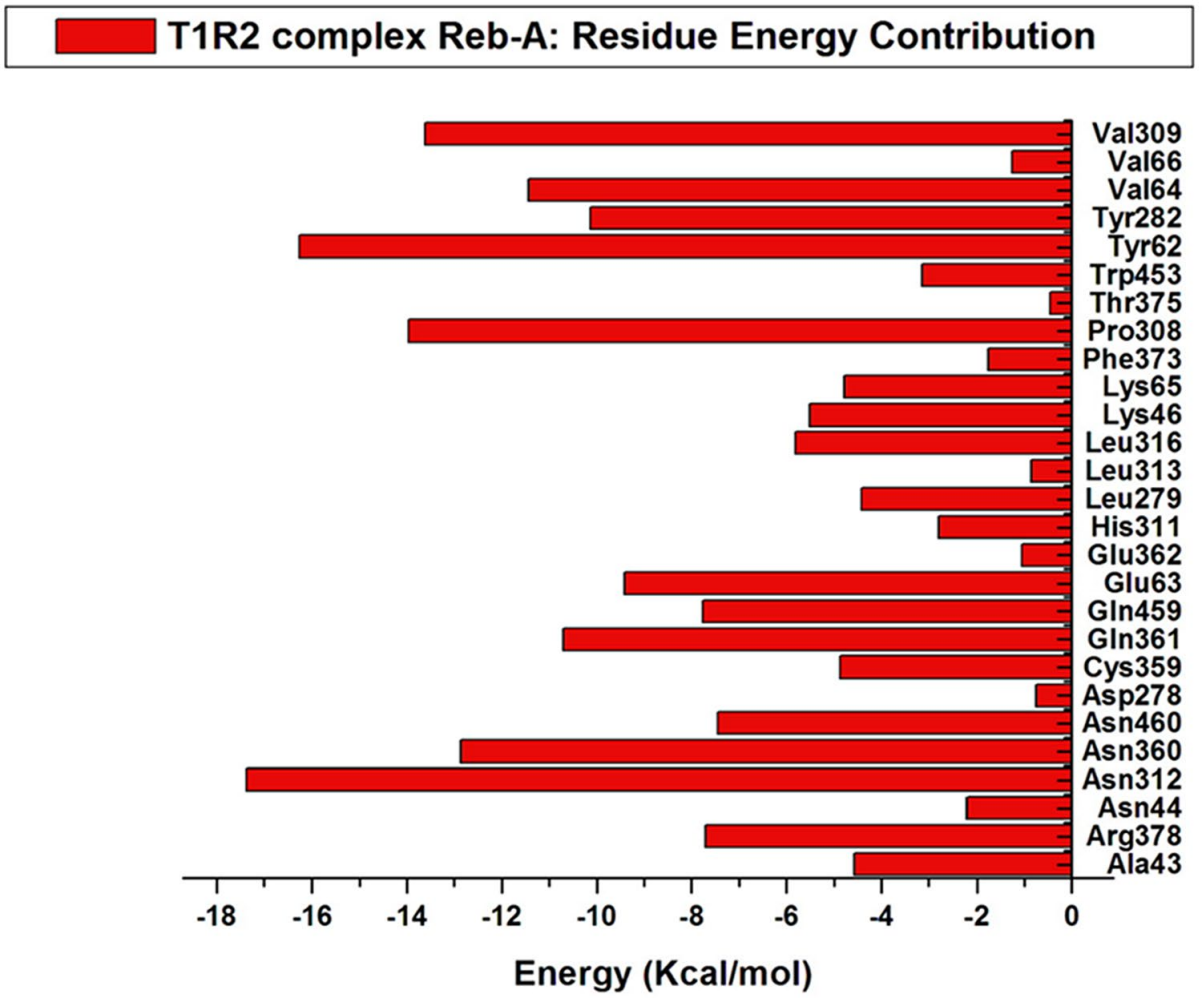
Per residue energy contributions of the active site residue to the T1R2-Reb-A complex generated on the Origin data analysis tool v6.0 (https://www.originlab.com/).

The hydrophobic interactions are located around the insoluble region of the Reb-A while the hydrogen bonds are formed in the most soluble regions. In addition, π–π stacking interactions and non-covalent bonds between the two aromatic rings have been observed in the respective residues Leu313–Leu316, Leu279–Tyr282, and Thr375–Arg378. The contribution of each of the active site residues in terms of their energy contribution are depicted in Fig. 5.

Likewise, the major energy contributions (kcal/mol) were observed from hydrophobic residues such as Tyr62 (− 16.265), Pro308 (− 13.969), Val309 (− 13.612), and polar residue such as Asn312 (− 17.100) and Asn360 (− 12.864).

Thereafter, the structural dynamics of the Reb-A complexed with the human T1R2 homolog was analysed by performing molecular dynamics simulations^83–87^ and free binding energy calculations^88,89^, which have proved to be very useful and successful in understanding the molecular basis of small molecules interacting with different biological targets including GPCR agonists^90,91^ and sweet taste receptors^86,87^. An MD simulation provides useful structural and energetic information about the interaction between the inhibitors (or molecules) and the target receptor. Studies have reported the interaction of steviol glycosides with human taste receptors experimentally^92^ and from a modelling perspective^16,93,94^. As previously reported in a study by Zhang et al.^95^, the Reb-A binds with the hinge region of the T1R2 venus flytrap domain. Foremost, the template chosen by Zhang et al. has low overall sequence identity of T1R2 to mGluR1 (~ 30%), yet, this study showed significant binding activity by residues closer to the hinge side of the T1R2 receptor. In this study, Reb-A binds to the human T1R2 homolog at the hinge region, very close to the V-shaped domain (discussed in Zhang et al.). As there is little information about the precise binding site of the human T1R2 experimentally, it is believed that the reported binding site by Zhang F. et al*.*, with slight variation in the angle of the active site residues, further support the findings observed in the current study.

### Molecular dynamics (MD) simulations analysis

In the present study, MM/GBSA was applied as it has been demonstrated to be more efficient for protein-drug systems^96–98^, carbohydrates^99^ and nucleic acids^100^. It utilizes a fully pairwise potential that is useful for decomposing the total binding free energy into atomic or group contributions^101^. This method explores the type of established interactions, calculating separately the components of the internal energy, the interaction energy and the free energy of Gibbs of solvation^102^. For the analysis of the binding free energy, 100 snapshots at a time interval of 10 ps were extracted from 100 ns of the MD trajectories. The system showed high interaction as evident from its binding affinity value (− 8.7 kcal/mol) and its free binding energy of − 60.44 kcal/mol.

**Figure 6:**
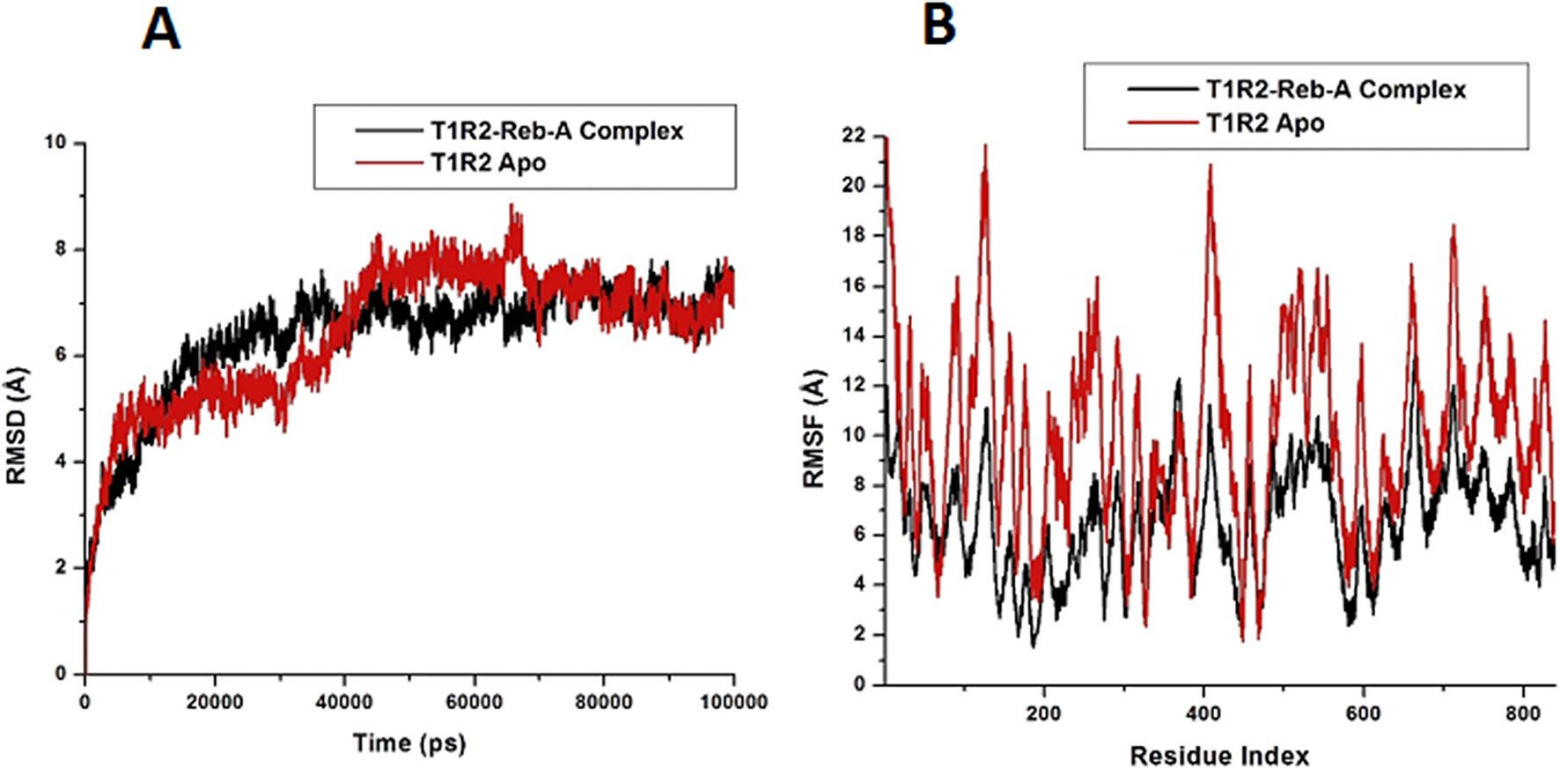
Root Mean Square Deviation (RMSD) and Root mean Square Fluctuation (RMSF) plots of the apo and bound T1R2 complex generated on the Origin data analysis tool v6.0 (https://www.originlab.com/).

In addition, the system stability and overall convergence of simulations were monitored in terms of Root Mean-Square Deviation (RMSD) and fluctuations of the human T1R2 backbone atoms. The RMSD and enabled us to verify that equilibration was achieved as illustrated in Fig. 6A. Similarly, the Root Mean Square Fluctuations (RMSF) of the average structure from the trajectory were computed to assess if the simulation results are in accordance with the crystal structure, and the results are shown in Fig. 6B.

The RMSD plots for T1R2 Apo and T1R2-Reb-A-complex shown in Fig. 6A, illustrates slight variations > 2 Å, confirming the stability of the equilibration after 40 ns. At approximately 70 ns, both systems reached a plateau convergence, which suggests that the delay experienced by the complex was due to the presence of the Reb-A. Overall, this plot showed the stability of the human T1R2 backbone.

Similarly, the RMSF plots shown in Fig. 6B indicate the temperature profiles of each amino acid residue. The RMSF plot of both apo and bound show a similar trend in the fluctuations of the amino acid residues. While it may be suggested that some residues were more mobile than the rest, probably due to the absence of a ligand in the apo, the T1R2-Reb-A complex demonstrated a stable structure upon binding.

**Figure 7:**
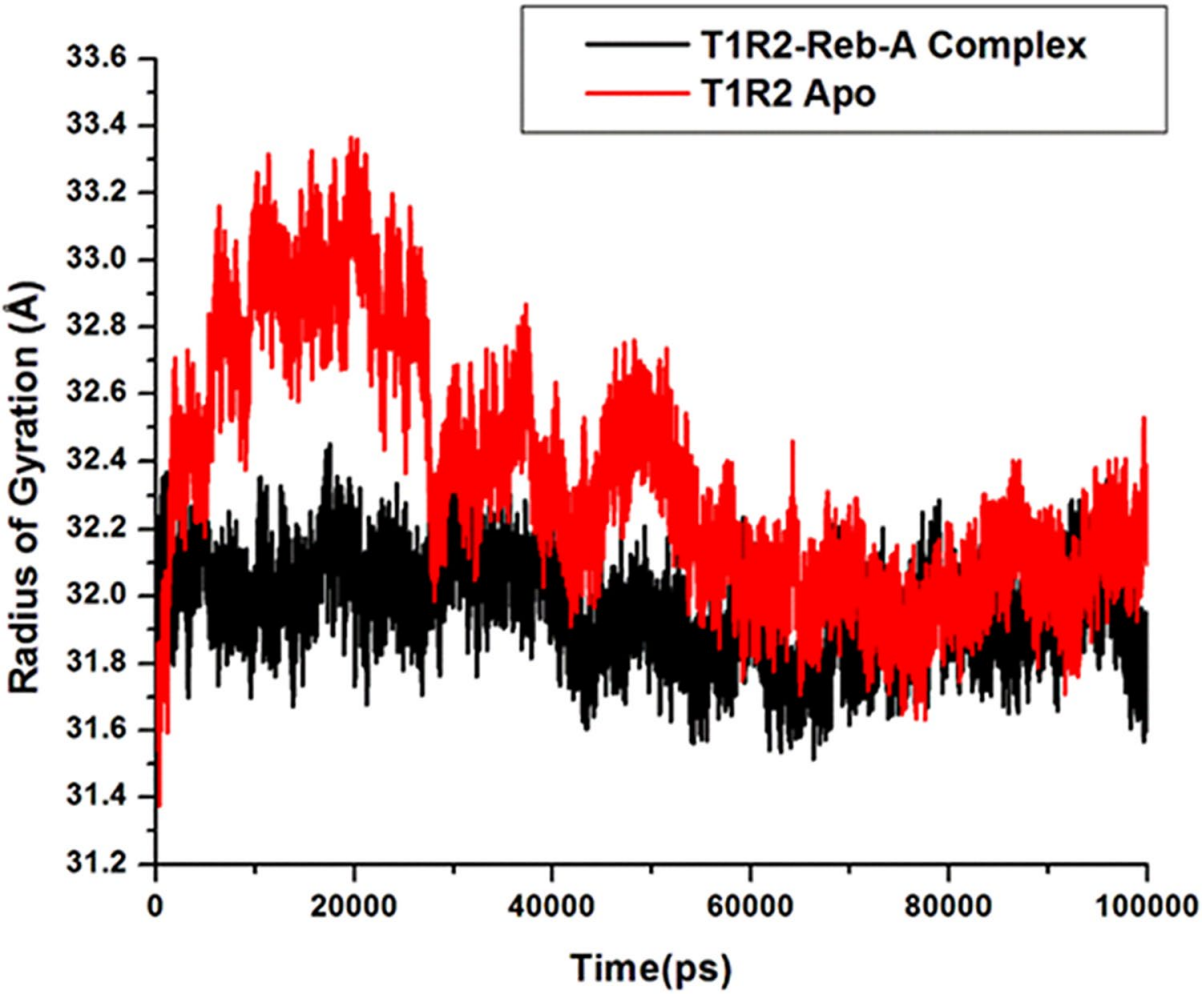
Radius of Gyration (Rg) plot for the apo and bound T1R2 complex generated on the Origin data analysis tool v6.0 (https://www.originlab.com/).

The radius of gyration (Rg), which studies the kinetics and thermodynamics of protein folding, is an indicator of the compactness of a protein and the stability of biomolecular structures^103^, was calculated for the T1R2-Reb-A complex. Topology of the Cα backbone has been found to play a role in protein folding^104^. Therefore, to measure the overall protein folding/unfolding and the compactness of the T1R2 protein, Rg change over time was analysed (see Fig. 7).

The average Rg of apo and bound T1R2 were compared and the overall Rg difference was observed between 5 and 100 ns for both systems are illustrated in Fig. 7. On average, the apo conformation showed an Rg value of 32.33 Å and 31.96 Å for the bound conformation. This explains the less compactness of the apo conformation of the T1R2. Generally, a decrease in Rg over time, as demonstrated by the T1R2-Reb-A complex, showed the stability of the system and its compactness. This indicates that the T1R2 demonstrates structural/conformational stability when bound to Reb-A. Generally, the RMSD, RMSF and the Rg indicate the conformational stability of the T1R2 homolog and the stability of the T1R2-Reb-A complex.

Following an in-depth study of the interaction of the T1R2-Reb-A complex, the second aspect of this work deals with their biomolecular interactions for sensing applications. The performance of these materials is often dependent on the properties of the interface, i.e. the degree of interaction with biomolecules at a molecular or atomistic level^105^. Several studies have also reported the role of nano surfaces in protein adsorption^106–112^.

### Materials modelling

All geometry optimized structures of the individual nanomaterials used in this study along with the energy values are tabulated in Table 1.

**Figure 8:**
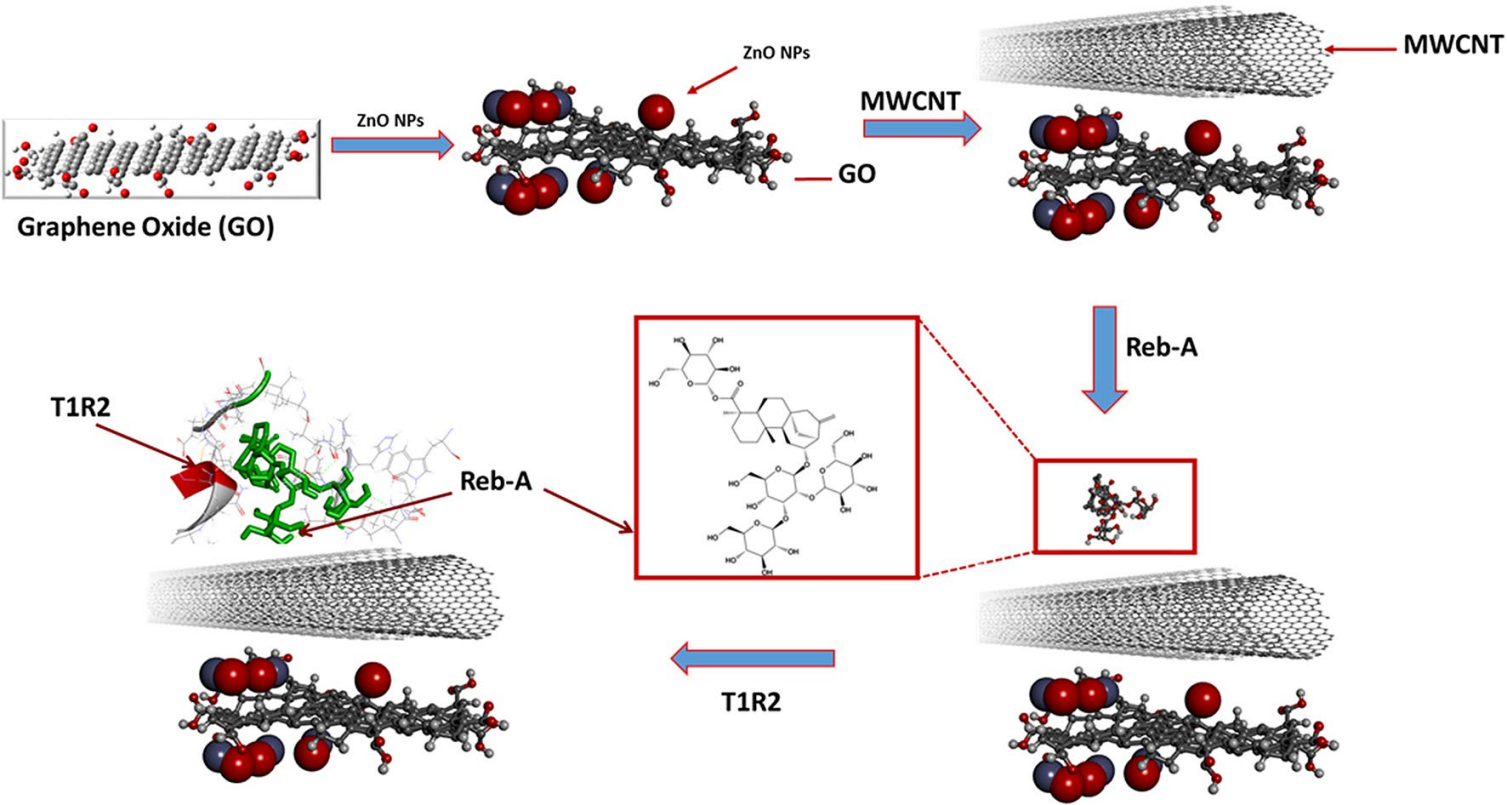
The steps followed in the modelling of GO/MWCNT/ZnONPs with MWCNT.

In line with the second key objective of this study, the performance of the interaction of the human T1R2-Reb-A complex on the surface of a (GO/ZnONPs) nanocomposite in the presence of MWCNT was assessed. Accordingly, a layer-by-layer approach was implemented with the interaction of GO/MWCNT/ZnONPs/T1R2-Reb-A described in Fig. 8 and for comparative purposes, the interaction in the absence of MWCNT for GO/ZnONPs/T1R2-Reb-A.

### Modelling of GO/MWCNT/ZnONPs with MWCNT

**Figure 9:**
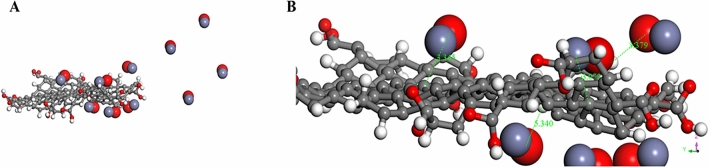
(**A**) ZnONPs adsorbed onto GO. (**B**) Zoomed in representation of the interaction between the closest ZnONPs to the GO surface. ∆G =  − 13.852 kcal/mol (Images generated on Materials studio^74^ license accessed through CHPC, Cape Town).

Using the optimized structures in Table 1 above, the modelling for Fig. 8 was undertaken in a stepwise fashion, with the ZnONPs adsorbed onto the surface of the GO, resulting in an adsorption energy of the ZnONP/GO complex (see Table 2) stabilized at − 13.852 kcal/mol. The adsorption performance and sensitivity of ZnONPs have been recently reported in literature^113,114^. As much as this study attempted to mimic experimental conditions, it is evident in Fig. 9A that only some of the ZnONPs take part in the interaction while others were a couple of angstroms away from the GO.

**Table 2:** Layer-by-layer system with multiwalled carbon nanotube (MWCNT) along with the calculated adsorption energies for Scheme 1.

Nano-systems	∆G (adsorption energy) kcal/mol
GO/ZnONPs	− 13.852
GO/MWCNT	− 170.447
GO/MWCNT/ZnONPs	− 76.642
GO/MWCNT/ZnONPs/T1R2-Reb-A	− 44.286

**Figure 10:**
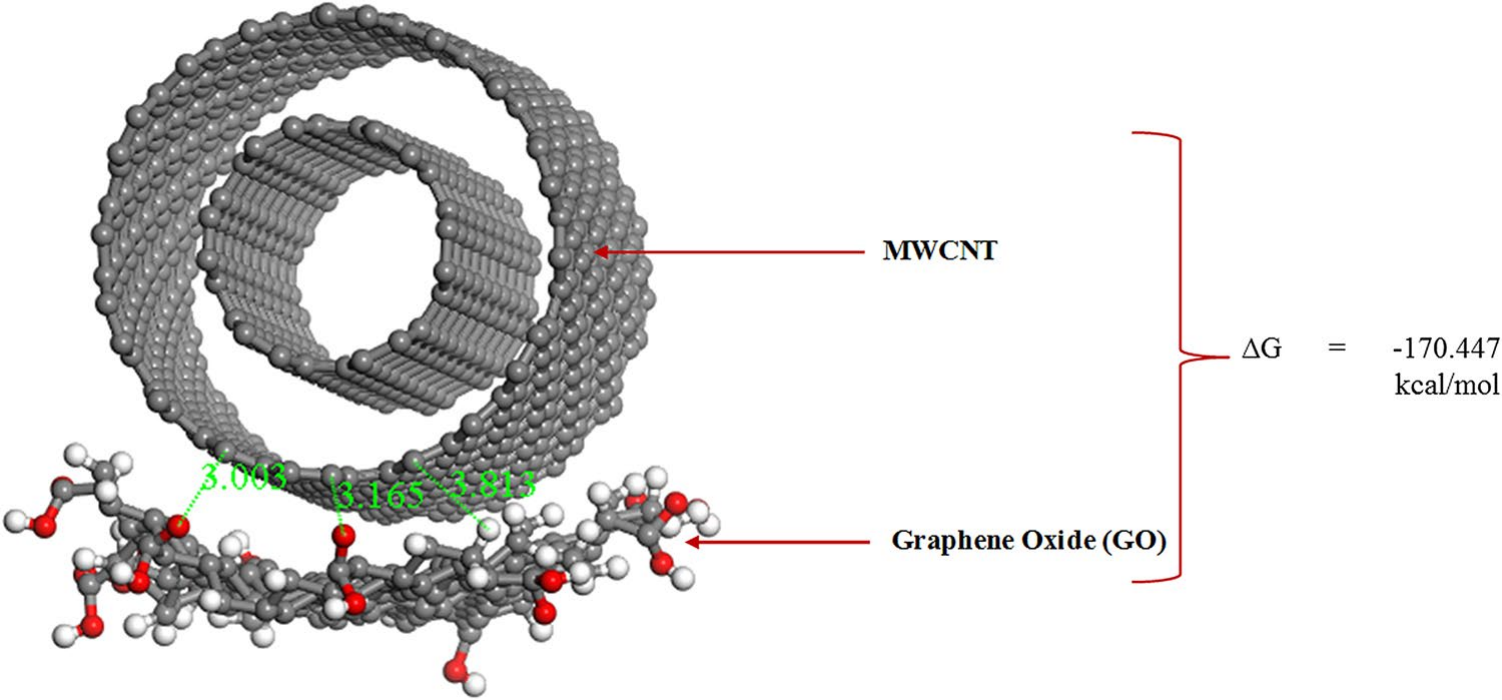
The physical adsorption of MWCNT onto GO. ∆G =  − 170.447 kcal/mol (Image generated on Materials studio^74^ license accessed through CHPC, Cape Town).

On closer inspection of Fig. 9B, it is clear that the inter-atomic distances between the ZnONPs and GO ranges between 3.072 and 5.340 Å. However, the modification of GO with MWCNT resulted in a considerable stabilization of the GO/MWCNT-complex as shown in Fig. 10.

**Figure 11:**
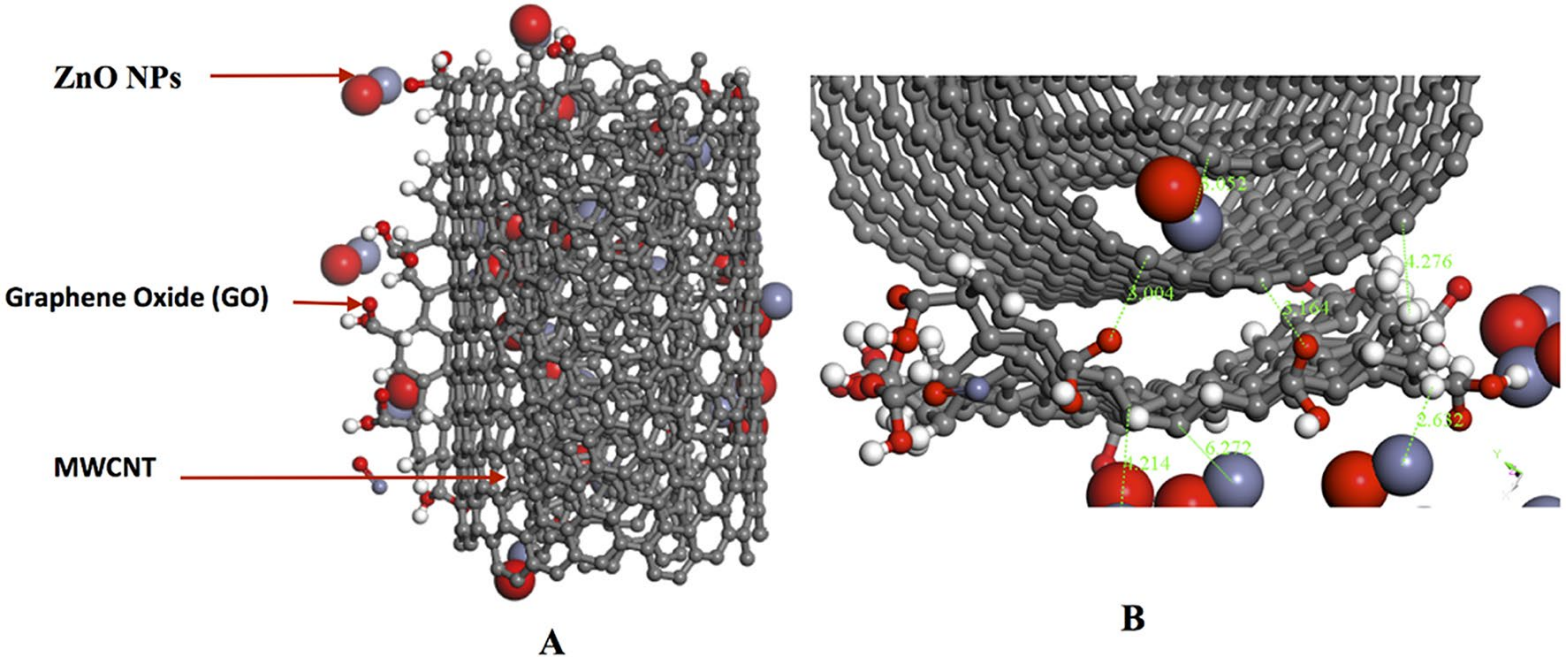
(**A**) Top view of the adsorption of MWCNT and ZnONPs on Graphene oxide. (**B**) A zoomed in representation of the adsorption of MWCNT and ZnONPs on Graphene oxide showing the distances between ZnONPs, MWCNT, and Graphene oxide. ∆G =  − 76.642 kcal/mol (Images generated on Materials studio^74^ license accessed through CHPC, Cape Town).

According to Fig. 11, the atom–atom distances within 3.003 Å and 3.813 Å were measured with respect to the nearest atoms from MWCNT and GO surface. These observed interatomic distances for adsorbent–adsorbate interaction is comparable to recent theoretical^115^ and experimental investigations on the adsorption performance of GO^116^ and functionalised MWCNT^117^, wherein the result of the FTIR spectra established the presence of several functional groups. Farghali et al.^117^ reported that numerous chemical sorption sites on MWCNTs surface could have been due to these functional groups. This further validated the reason behind the high adsorption energy of GO/MWCNT in the current study (∆G =  − 170.447 kcal/mol).

Figure 11 illustrates a further adsorption with the ZnONPs and the MWCNT with an energy value of − 76.642 kcal/mol.

According to Fig. 11A, the surface area of the GO was adequately utilised by the MWCNT and ZnONPs. As depicted in Fig. 11B, the shortest atom–atom distance between ZnONPs and GO was 2.632 Å. In comparison, the distance between the closest atoms of ZnONPs and MWCNT was 3.052 Å, while it was 3.004 Å for GO and MWCNT. The large difference between the adsorption energy when ZnONPs are included (∆G =  − 76.642 kcal/mol) and excluded (∆G =  − 170.447 kcal/mol) could be due to a better interaction of the ZnONPs at the surface of the GO. On a closer inspection of Fig. 11B, it is noticeable that the ZnONPs are adsorbed more on the side where MWCNT is absent. To find a suitable explanation for this, another calculation was carried out to provide an insight into the adsorption of ZnONPs and MWCNT only, resulting in an adsorption energy of -18.458 kcal/mol.

**Figure 12:**
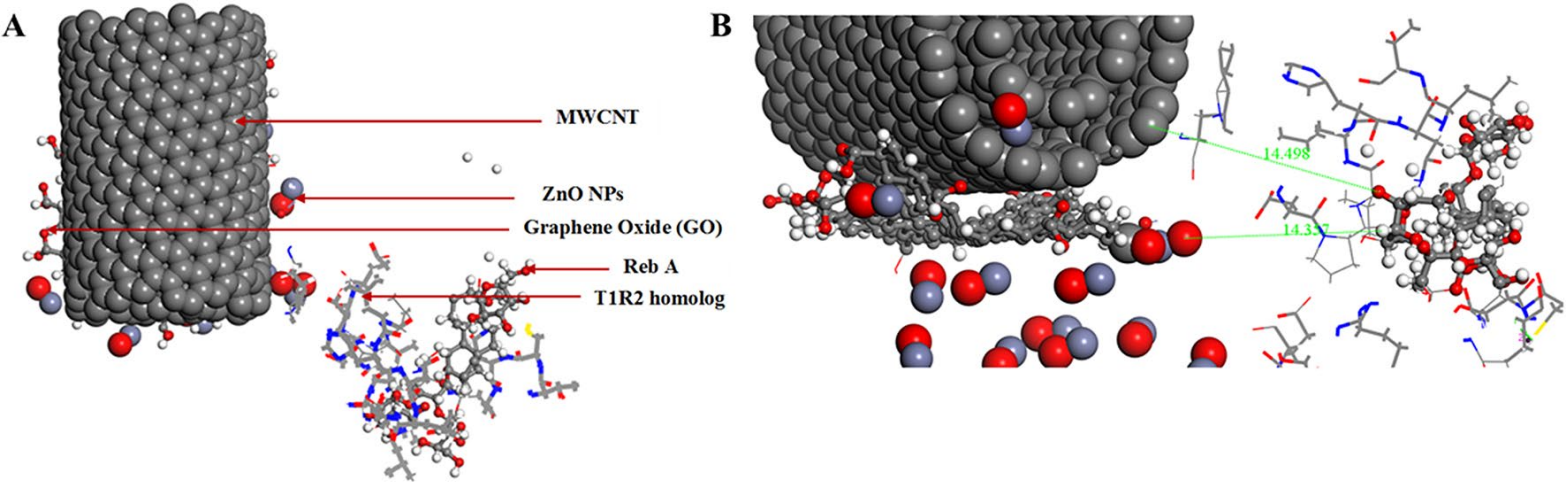
(**A**) Top view of the adsorption of the MWCNT, ZnO nanoparticles, and human T1R2 homolog-Reb-A complex onto graphene oxide surface. (**B**) Side view of showing the distances of the Reb-A to the MWCNT and ZnONPs respectively. ∆G =  − 44.286 kcal/mol (Images generated on Materials studio^74^ license accessed through CHPC, Cape Town).

Figure 12 illustrates the interaction of the MWCNT, ZnONPs and human T1R2-Reb-A complex onto the graphene oxide surface, with an adsorption energy of − 44.286 kcal/mol. When Reb-A was adsorbed onto the existing layer of GO/MWCNT/ZnONPs, the shortest atom–atom distance between GO and Reb-A was 2.72 Å while for GO and MWCNT, a distance of 5.024 Å was reported between the nearest atoms. The Reb-A seem to propel itself to interact more with the MWCNT by folding alongside its plane.

Figure 12A shows the top view of the overall interaction study (see Fig. 8) while Fig. 12B shows the side view along with the distances between the Reb-A and GO (14.357 Å); and Reb-A-MWCNT (14.498 Å). It is evident from the adsorption energy (∆G =  − 44.286 kcal/mol) that more hydrophobic interaction exists between the biomolecular part (T1R2 homolog and Reb-A) as opposed to the nanomaterials part. This change in ∆G could be because of intermolecular interactions i.e. when highly hydrophobic molecules interact with each other, the enthalpy increases because some of the hydrogen bonds will be broken to form an O–H group with the other molecule. That is why a high binding affinity and free binding energy values were reported for the human T1R2 homolog-Reb-A in this study. Thus, it is safe to say that the reduction in ∆G, observed in Fig. 12, is as a result of the more hydrophobic interaction between the human T1R2 homolog and Reb-A described earlier in Fig. 4. The performance of MWCNT when immobilised onto nanocomposites have been studied in different systems and have been reported to enhance the reactivity and/or the sensitivity of the systems studied^118–122^. Therefore, a key finding of this study, is the enhanced adsorption on the T1R2-Reb-A complex in the presence of MWCNT.

**Figure 13:**
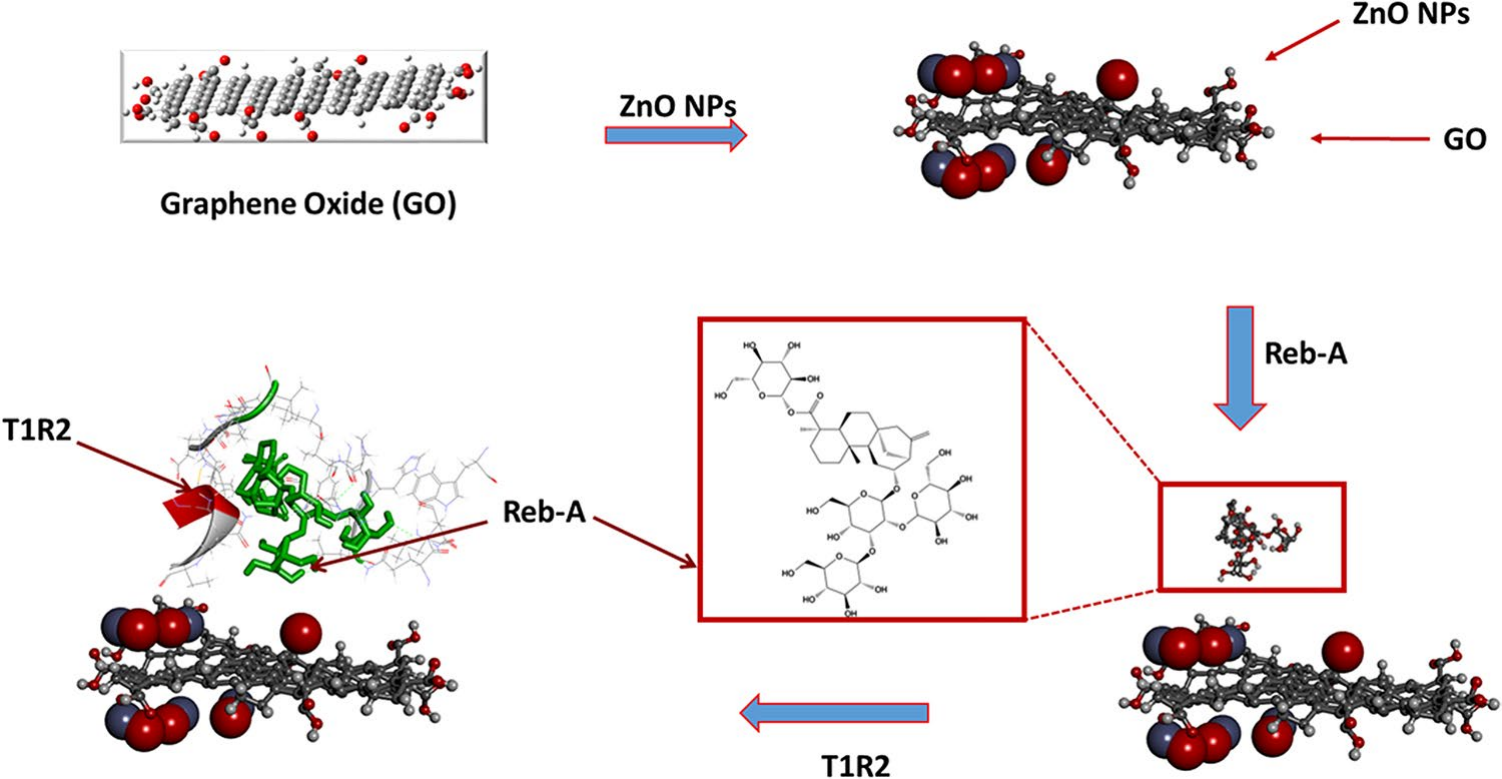
The steps followed in the modelling of GO/ZnONPs without MWCNT.

For comparative purposes, the performance was tested in the absence of MWCNT as proposed in Fig. 13 below.

### Modelling of GO/ZnONPs without MWCNT

**Figure 14:**
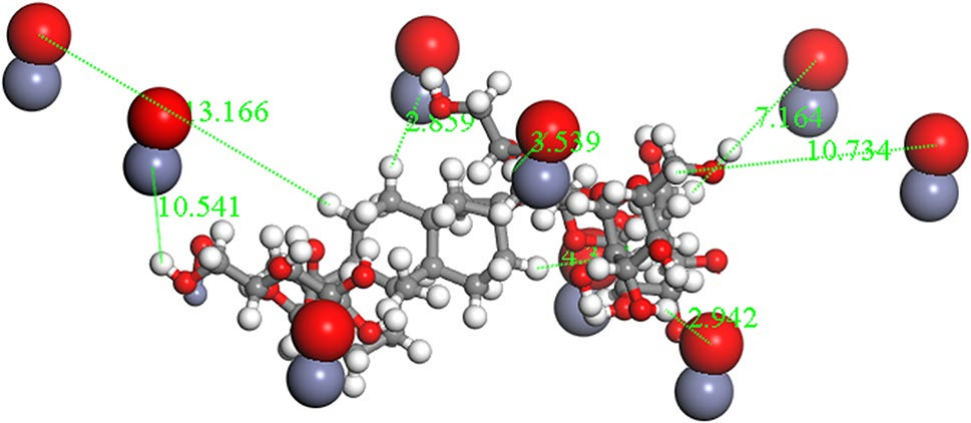
Adsorption of Reb-A onto ZnONPs. Adsorption energy =  − 16.725 kcal/mol (Image generated on Materials studio^74^ license accessed through CHPC, Cape Town).

Using the optimized structures in Tables 1 and 3, the modelling for Fig. 13 was undertaken in a similar stepwise fashion as described in Fig. 8, except in the absence of MWCNT. Figure 14 illustrates the adsorption of Reb-A onto ZnONPs.

**Table 3:** The layer-by-layer system without multiwalled carbon nanotube (MWCNT) along with calculated adsorption energies (kcal/mol).

Nano-system	∆G (adsorption energy) kcal/mol
ZnONPs/Reb-A	− 16.725
GO/ZnONPs/T1R2-Reb-A	− 38.328

**Figure 15:**
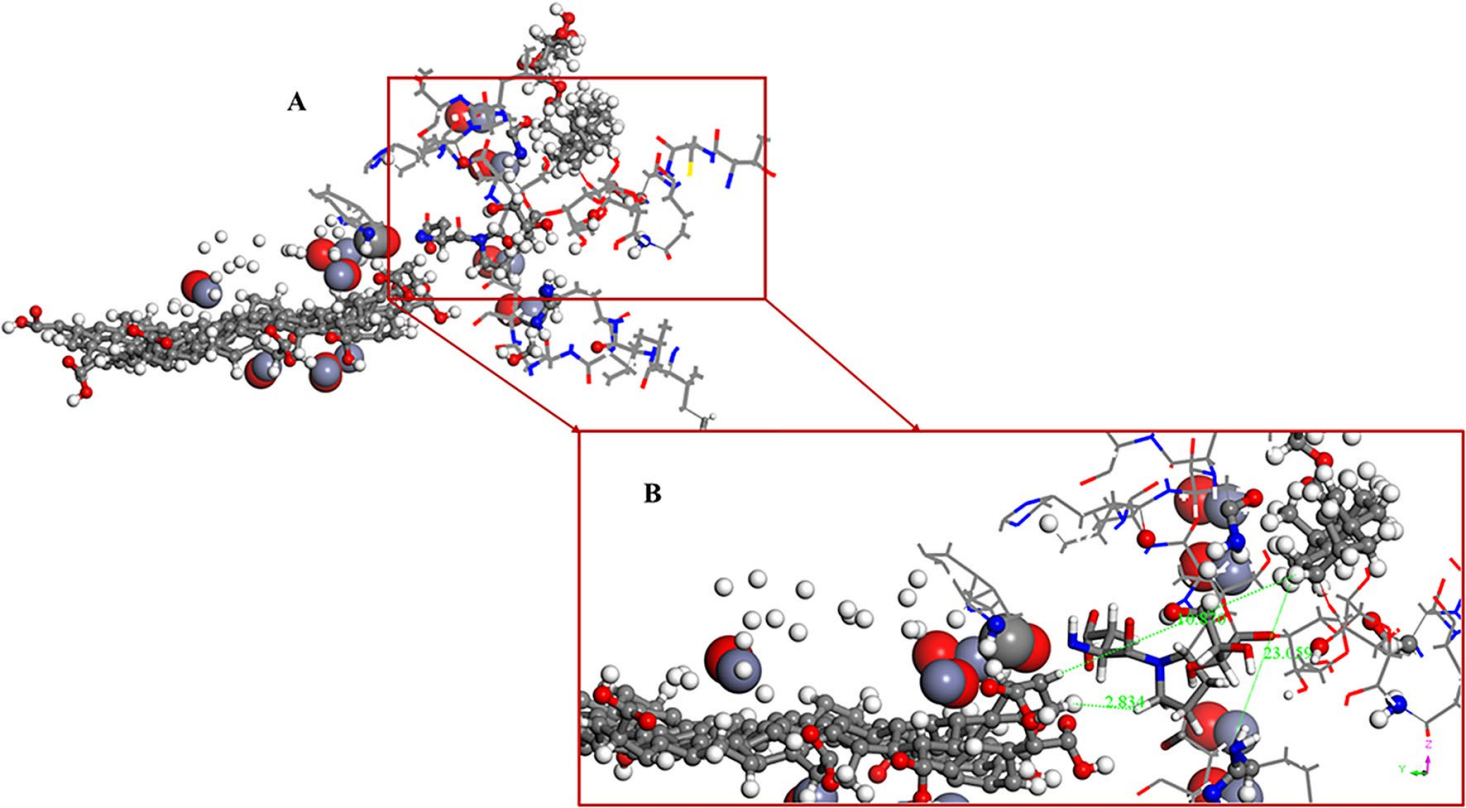
The adsorption of ZnONPs, T1R2-Reb-A complex onto GO surface ∆G =  − 38.328 kcal/mol (Image generated on Materials studio^74^ license accessed through CHPC, Cape Town).

There was an increase in adsorption energy when Reb-A interacted with the ZnONPs (Fig. 14), resulting in an adsorption energy of − 16.725 kcal/mol. This could be due to the formation of a hydrogen bond interaction between the H on the Reb-A and the O on the ZnONPs. Figure 15 further illustrates the ZnONPs adsorbed onto GO followed by direct adsorption of the T1R2-Reb-A complex onto the GO surface.

According to Fig. 15A, a good bio-molecular interaction exists between the human T1R2 and Reb-A, which indicates an enhanced sensitivity of Reb-A in the presence of the human T1R2. Furthermore, Fig. 15B better explains the inter-atomic distances of Reb-A to the GO (− 10.870 Å) and the ZnONPs (23.059 Å) respectively.

Therefore, this study emphasises the role of nanomaterials in the layer-by-layer adsorption and we could say that the different surface arrangements of the nanomaterials play a vital role in its interaction pattern with the T1R2-Reb-A complex.

### Comparison of experimental data with computational results

Differential pulse voltammetry (DPV) is an analytical tool comprising of advantages such as short pulse time and sensitivity for both quantitative analysis and understanding chemical reaction kinetics, thermodynamics, and mechanisms. In this study, due to its sensitivity, DPV was used to accurately detect the interaction of Reb A at T1R2/GO/Pt and T1R2/MWCNTs/Pt under optimized electrochemical parameters (pH 11.0, scan rate: 0.09 V s^−1^, and deposition time: 40 s).

**Figure 16:**
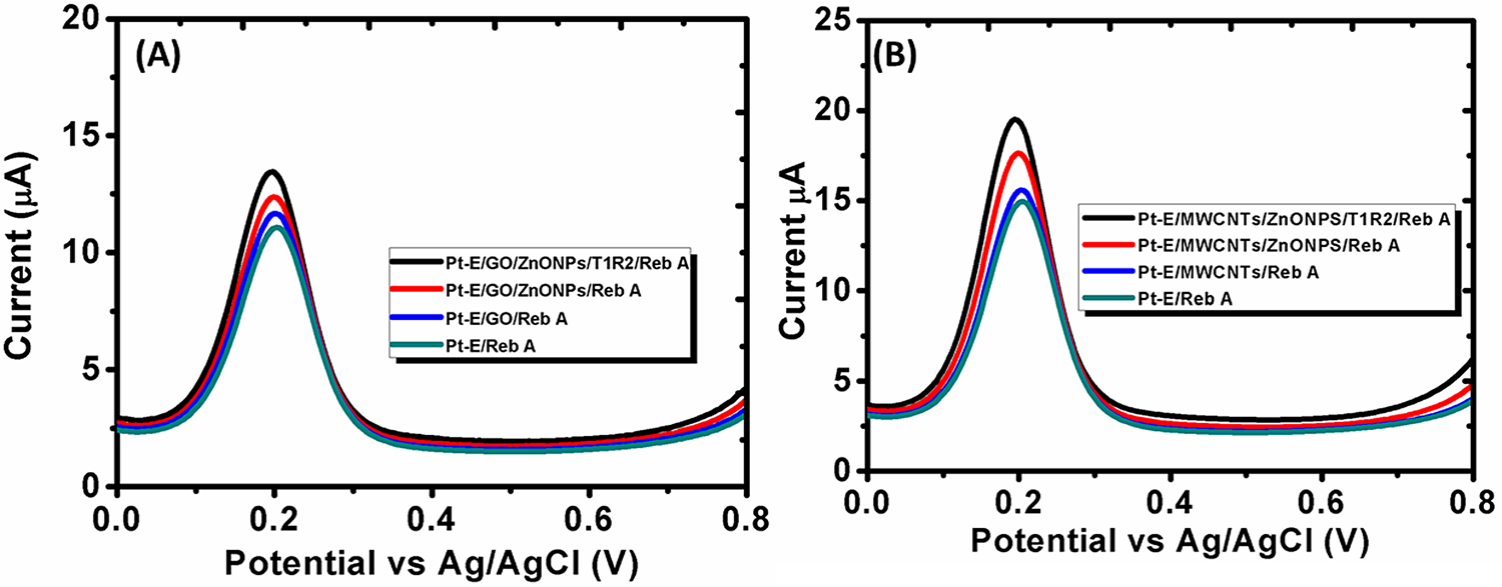
DPV of Reb A at pH 11.0 borate buffer using (**A**) T1R2/GO/Pt and (**B**) T1R2/MWCNTs/Pt immunosensor.

Figure 16A,B displays obtained anodic peak currents with T1R2/GO/Pt and T1R2/MWCNTs/Pt at 0.2 V vs Ag/AgCl with a linear relationship between anodic currents. It was demonstrated that the peak currents obtained with T1R2/MWCNTs/Pt were greater compared to T1R2/GO/Pt, indicating the rapid electron transfer capability and higher surface area of MWCNTs. The modelled MWCNTs demonstrated similar trends in terms of adsorption energies where the ΔG of the systems with MWCNTs was higher compared to systems without MWCNTs.

## Conclusions

This study present insights into the interaction between biomolecules at the nanostructure interface by exploring the modification of GO/MWCNT with ZnONPs and its application as an adsorbent for the human T1R2-Reb-A complex from both computational and electrochemical perspectives. Various theoretical calculations and analysis assisted in the understanding of the mechanism of bio-molecular interaction and adsorption at the molecular level through the estimation and comparison of different properties of the two systems (with and without MWCNT). This layer-by-layer adsorption mechanism allows for the structural elucidation for systems modified with and without MWCNT, in which the former adsorbent showed a better performance than the latter. For all the systems studied, the presence of MWCNT showed higher adsorption capacities in systems modified with MWCNT as opposed to unmodified systems. In addition, the bio-molecular interaction between T1R2-Reb-A complex showed higher binding affinity as well as higher free binding energy. Also, hydrophobic interactions played a major role in the interaction between Reb-A and the T1R2 homolog. Different metrics, such as the biomolecular interaction and the adsorption at the materials interface, used in this study provided the best explanation for the overall adsorption mechanism. The high adsorption capacity reported in this study are in agreement with previously reported experimental work^117^. Comparing the adsorption energies of the two systems, GO/MWCNT/ZnONPs/T1R2-Reb-A (∆G =  − 76.642 kcal/mol) shows a better adsorption energy due to the presence of MWCNT in contrast to GO/ZnONPs/T1R2-Reb-A (∆G =  − 13.852 kcal/mol), which is a key finding of this work. The obtained experimental results were in good agreement with our reported computational results in terms of adsorption energies. This study demonstrates the usefulness of a layer-by-layer strategy to assess the performance of the T1R2-Reb-A complex by the incorporation of MWCNT with enhanced selectivity, sensitivity and robustness of the proposed immunosensor.

### Future work

The obtained modelling results of T1R2 and Reb A on GO as well as MWCNTs surface has given new insights in choosing T1R2/T1R3 subunit as a new biomarker for the development of additional electrochemical immunosensor that could result in more opportunities in terms of enhanced selectivity in quality control laboratories in the food beverage industries.

## Supplementary information

Supplementary Information.
