# Supplementary material for: An in-silico layer-by-layer adsorption study of the interaction between Rebaudioside A and the T1R2 human sweet taste receptor: modelling and biosensing perspectives
Source: Sci Rep. 2020 Oct 27;10:18391. doi: 10.1038/s41598-020-75123-4 (PMC7591876; doi:10.1038/s41598-020-75123-4)
Supplement: Supplementary file 1 — Supplementary Information. [file 41598_2020_75123_MOESM1_ESM.docx]

**An in-silico layer-by-layer adsorption study of the interaction between Rebaudioside A and the T1R2 human sweet taste receptor: modelling and biosensing perspectives**

Olayide A Arodola^1^, Suvardhan Kanchi^1^, Phathisanani Hloma^1^, Krishna Bisetty^1^, Abdullah M. Asiri^2^ and Inamuddin^2^

^1^Department of Chemistry, Durban University of Technology, P.O Box 1334, Durban 4000, South Africa. ^†^Correspondence: bisettyk@dut.ac.za, Olayide.arodola@gmail.com

^2^Chemistry Department, Faculty of Science, King Abdulaziz University, Jeddah 21589, Saudi Arabia Corresponding email address: inamuddin@zhcet.ac.in

**Supplementary Material**

**T1R2 homology model Ramachandran plot and outliers.**

**
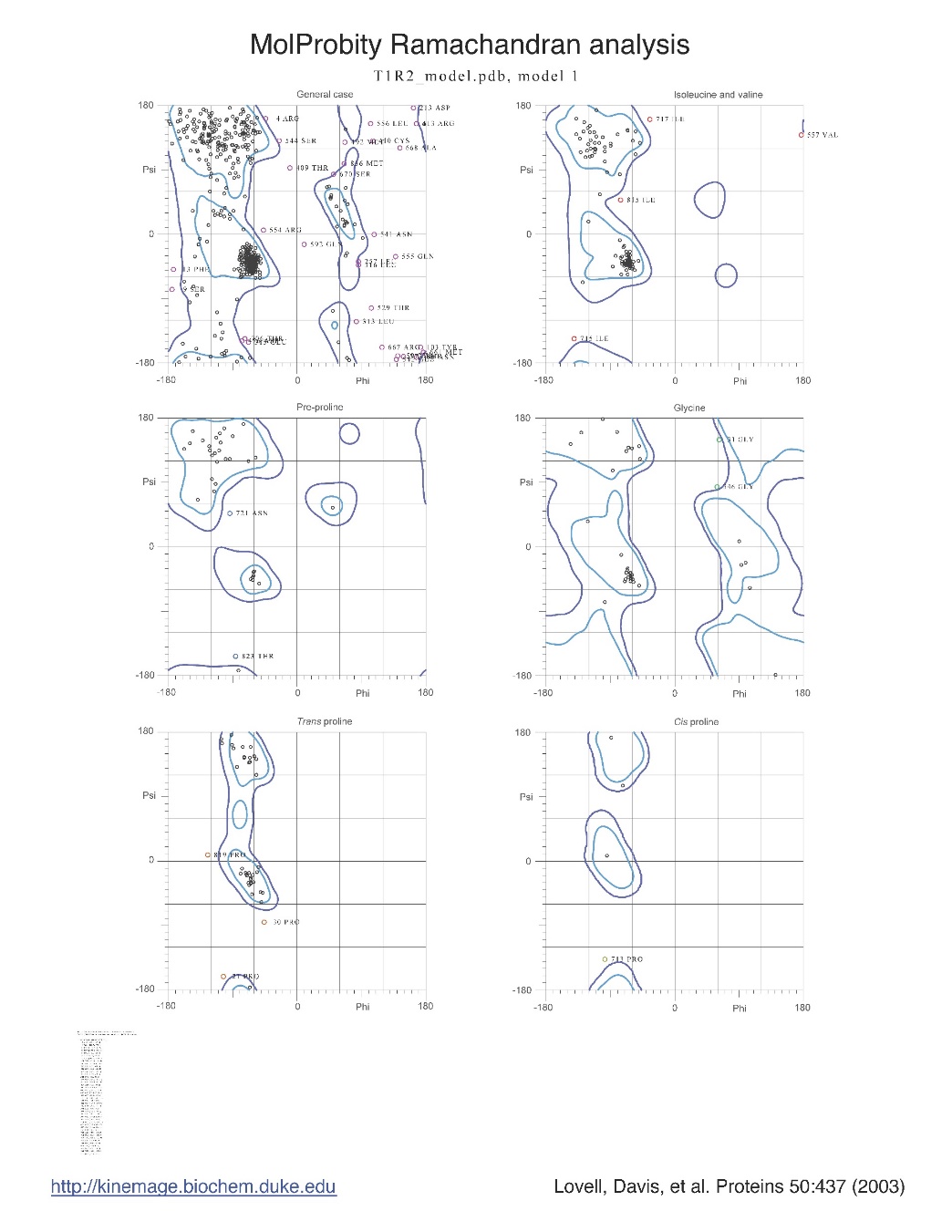
**

There were 43 outliers (phi, psi):

4 ARG (-44.3, 162.9)

9 SER (-175.7, -77.7)

13 PHE (-173.5, -49.5)

21 PRO (-103.6, -161.6)

30 PRO (-46.2, -86.0)

31 GLY (62.7, 150.2)

59 CYS (142.0, -170.7)

103 TYR (174.0, -158.4)

213 ASP (163.0, 177.4)

257 LEU (86.1, -38.0)

313 LEU (83.6, -122.1)

315 GLU (-68.2, -151.3)

316 LEU (86.9, -43.5)

346 GLY (59.2, 84.8)

368 ASN (167.7, -172.1)

373 PHE (149.2, -172.0)

409 THR (-10.6, 93.8)

410 CYS (106.7, 131.7)

413 ARG (167.3, 155.6)

456 ASP (-77.9, -149.1)

492 MET (67.8, 129.2)

529 THR (105.0, -103.1)

541 ASN (108.6, 0.2)

542 GLU (139.7, -176.0)

544 SER (-25.3, 131.9)

554 ARG (-47.6, 6.6)

555 GLN (138.8, -31.3)

556 LEU (103.6, 155.7)

557 VAL (177.4, 139.4)

592 GLN (10.0, -14.9)

661 MET (177.1, -165.5)

667 ARG (119.8, -158.8)

668 ALA (144.2, 121.6)

670 SER (51.2, 84.7)

706 THR (-73.4, -146.9)

713 PRO (-98.8, -137.9)

715 ILE (-141.3, -146.5)

717 ILE (-35.5, 161.8)

721 ASN (-94.4, 47.6)

815 ILE (-77.0, 48.5)

819 PRO (-126.0, 9.6)

823 THR (-86.1, -153.8)

836 MET (66.2, 99.1)
